# Scale of attitudes toward alcohol - Spanish version: evidences of validity and reliability^1^

**DOI:** 10.1590/1518-8345.1721.2918

**Published:** 2017-08-03

**Authors:** Erika Gisseth León Ramírez, Divane de Vargas

**Affiliations:** 2 Doctoral student, Escola de Enfermagem, Universidade de São Paulo, São Paulo, SP, Brazil. Scholarship holder at Coordenação de Aperfeiçoamento de Pessoal de Nível Superior (CAPES), Brazil.; 3 PhD, Associate Professor, Escola de Enfermagem, Universidade de São Paulo, São Paulo, SP, Brazil.

**Keywords:** Health Knowledge, Attitudes, Practice, Psychological Tests, Validity of Tests, Reproducibility of Results, Alcoholism, Scale

## Abstract

**Objective:**

validate the Scale of attitudes toward alcohol, alcoholism and individuals with alcohol use disorders in its Spanish version.

**Method:**

methodological study, involving 300 Colombian nurses. Adopting the classical theory, confirmatory factor analysis was applied without prior examination, based on the strong historical evidence of the factorial structure of the original scale to determine the construct validity of this Spanish version. To assess the reliability, Cronbach’s Alpha and Mc Donalid’s Omega coefficients were used.

**Results:**

the confirmatory factor analysis indicated the good fit of the scale model in a four-factor distribution, with a cut-off point at 3.2, demonstrating 66.7% of sensitivity.

**Conclusions:**

the Scale of attitudes toward alcohol, alcoholism and individuals with alcohol use disorders in Spanish presented robust psychometric qualities, affirming that the instrument possesses a solid factorial structure and reliability and is capable of precisely measuring the nurses’ atittudes towards the phenomenon proposed.

## Introduction

The percentage of alcohol use in Latin America surpasses the global average (40%)^1^ ^-^ ^2^. These percentages entail countless health problems, putting this problem high on the regional Public Health agenda. This demands training and knowledge from the health professionals, who are increasingly confronted with this population in the different care contexts^3^. As the professionals’ knowledge on the problem is directly associated with their attitudes during care delivery to the users, it is important to identify their attitudes towards alcohol and aspects related to its use^4^.

Although attitudes have been largely defined since the 1960’s, some characteristics currently prevail as structural components, including the values, beliefs and feelings; and their importance as predictors of behaviors or actions towards a specific situation^5^. These components can be characterized as apparently immeasurable latent traits, but can be assessed by means of psychological measures, which permit evaluating and understanding their nature, causes and consequences in the different contexts, theoretical advancing in social psychology and its application to important behavioral changes in the study population^6^. 

An analysis of the tools available to measure the nurses’ attitudes towards individuals with alcohol use disorders reveals that most are available in English^7^ ^-^ ^8^, and were therefore developed in the Anglo-American social and cultural context. Only two of the instruments available to measure the nurses’ attitudes in that language have been translated to Spanish in Latin America and applied to populations in the same region^9^ ^-^ ^11^. Nevertheless, the validation processes of these tools are not available in the literature^9^, while other sources discuss these processes superficially^10^, which permits questioning the method adopted to verify the validity of the scales used thus far in Spanish.

Specialized institutes estimate that 423,252.042 people in Latin America speak Spanish. Concerning the proportion of nurses in that population, the Pan American Health Organization estimates that about 452,023 nurses are distributed across countries like Argentina, Bolivia, Chile, Colombia, Costa Rica, Cuba, El Salvador, Mexico, Nicaragua, Paraguay and the Dominican Republic. These considerations suggest that many nursing professionals in Latin America speak Spanish, which by itself indicates the need for a translated instrument with factorial structure evidence in Spanish, which complies with the international guidelines for these processes, aiming to guarantee the reliability of its use in that language.

Among the existing instruments in Latin America, the Scale of attitudes toward alcohol, alcoholism and individuals with alcohol use disorders, elaborated in Brazil in 2008^12^, stands out because it was elaborated in a country of Latin American culture, which puts it closer to the context of the target population for the instrument validation. On the other hand, as opposed to the scales used in the studies executed in the region, evidences have been published on its elaboration and refinement process, demonstrating robust psychometric qualities in its language or origin^12^ ^-^ ^14^. En este sentido, el presente estudio tiene por objetivo buscar evidencias de estructura factorial y confiabilidad de la Escala de actitudes frente al alcohol, al alcoholismo y a la persona con trastornos relacionados al uso de alcohol (EAFAA) en enfermeros hispanohablantes en el contexto colombiano. 

## Method

A methodological study was undertaken, involving nursing professionals affiliated with medium and large health institutions in the city of Bogota (Colombia) between November 2014 and April 2015. 

### Sample

To guarantee the quality of the psychometric analysis of the scale, in the calculation of the sample, at least five participants were guaranteed for each instrument item^15^. Initially, the sample consisted of 650 nurses from three hospitals (60%) and two (40%) primary health care services, 303 (46%) of whom returned the completed instrument. The following inclusion criteria were considered: being a nurse, professionally active at the time of the data collection. The sample predominantly included female (85%), single participants (49.5%), who informed that they had received some type of training to work in the field of alcohol and other drugs (57%).

### Instruments

To collect the data, a sociodemographic questionnaire was applied, which contained 14 questions, distributed between sociodemographic data: Age, marital status, sex; and questions related to the nurse’s educational background and experience in the theme alcohol and other drugs: specific training in the area, hour load during education, prior experience with individuals with alcohol use disorders; and, finally, the Scale of attitudes toward alcohol, alcoholism and individuals with alcohol use disorders, translated and validated to the Spanish language^16^. This scale was elaborated in Brazil in Portuguese, conceived based on the classical test theory (CTT) and the latent trait theory, consisting of 49 assertions grouped in four factors, which could be answered using a Likert-type scale with five alternative answers, ranging between (1) Strongly disagree and (5) Strongly agree.

### Data collection procedure

To collect the data, the nurses were contacted individually at work, in all shifts (morning, afternoon and night). After describing the research objective and guaranteeing that their identity would be preserved, they received a sealed envelope, which contained the informed consent, the sociodemographic questionnaire and the translated and adapted version of the EAFAA, including instructions for completion, establishing a maximum deadline to return the completed instrument within 24 hours.

### Data analysis procedure

The verification of the factorial structure of the EAFAA - Spanish version was based on the classical test theory (CTT), also used in the construction and validation of the original instrument. The theory explains the manifestation of a personality characteristic or latent trait by means of measuring tools, using statistical tests to demonstrate this representation. These include factorial analysis, internal consistency analysis, sensitivity and specificity analyses of the instrument. The collected data were stored in a Microsoft Excel database and analyzed in the software R (R Project for Statistical Computing) versão 3.0. 

The fitness of the data matrix for the application of factorial analysis was verified by means of the Kaiser-Meyer-Olkin (KMO) test, considering 0.50 as the minimum adequacy coefficient. As the original version of the EAFAA has been validated in multiple populations and has demonstrated a robust factorial structure^12^ ^-^ ^14^, the confirmatory factorial analysis technique was chosen to verify the proposed model. In line with the steps suggested in the literature, during the confirmatory factorial analysis, the adequacy ratese of the factorial model were calculated, using the weighted least squares estimation method and the correlation test between the factors (-1 ≤ r ≤ +1). For this calculation, the 49 items resulting from the content validation of the EAFAA - Spanish version were included in the model, with the prior imposition of a four-factor structure to maintain the analysis as consistent as possible with the original instrument. The minimum factor loading considered to maintain the items was 3.0, as indicated in the literature on this type of studies^17^.

To analyze the internal consistency of the scale and each of its four component factors, Cronbach’s alpha and Mc Donald’s Omega were used^18^. The sensitivity and specificity of the EAFAA were verified using the ROC curve technique^19^. For all statistical tests, a 5% confidence level was adopted.

### Ethical aspects

This study received approval from the research ethics committee of the University of São Paulo School of Nursing, under opinion 843.955\2014, and from each of the health institutions that participated in the data collection. In addition, the author of the EAFAA authorized the validation of the Spanish version.

## Results

**Figure 1:**
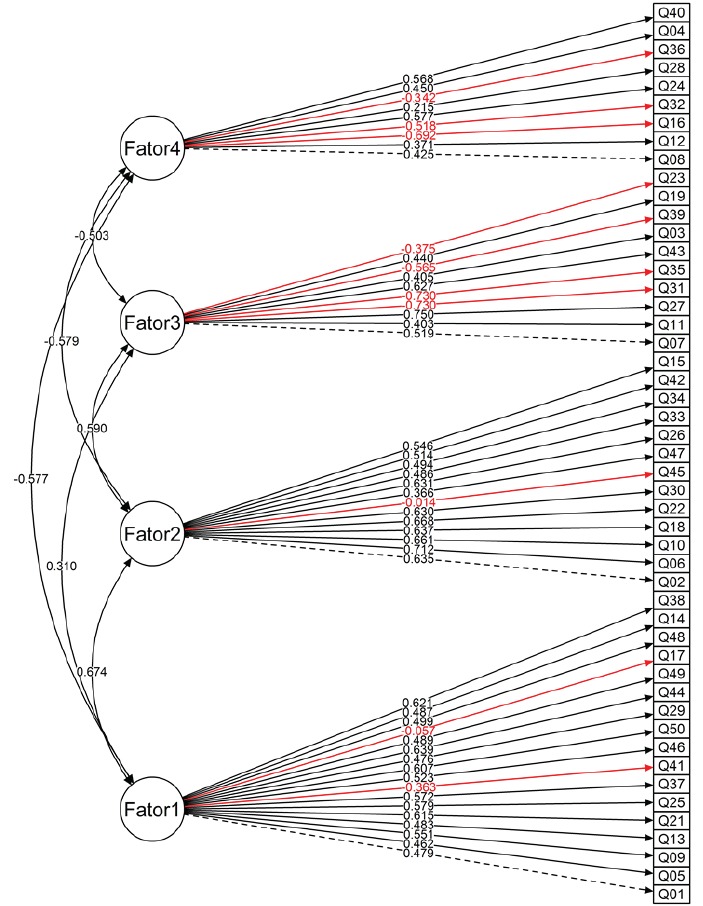
Path graph of confirmatory factorial analysis of EAFAA - Spanish version, Bogota (BOG), Colombia, 2015

The data matrix showed to be fit for the factorial analysis (FA), indicating a MO coefficient of 0.86. The adequacy rates of the factorial model appointed satisfactory coefficients (Table 1), suggesting the maintenance of the four-factor structure for the adapted version of the EAFAA, previously imposed in the original instrument. The path model presented in Figure 1 indicates the factor loadings of the items in the distribution per factor and the corresponding correlations.

**Table 1:** Adequacy evaluation of of the four-factor model of the EAFAA - Spanish version by weighted least squares estimation, Bogotá (BOG), Colombia, 2015

**Fit index**	**Result**
RMSEA*	**0.05**
**SRMR** ^**†**^	**0.007**
**Tucker - Lewis Index (TLI)**	**0.91**
**CFI** ^**‡**^	**0.92**

*Root Mean Square Error of Approximation

†Standardized Root Mean Square Residual

‡Comparative Fit Index

According to the analysis of the model and the preset exclusion criteria for the maintenance of the items (>0.3), we chose to eliminate item 45: “Individuals with alcohol use disorders cooperate with their treatment”, in view of a factor loading of 0.014. 

The correlation test between the factors of the EAFAA - Spanish version indicated significant mutual correlations, as represented in Table 2.

**Table 2:** Correlation test between the four factors of the EAFAA - Spanish version. Bogotá (BOG), Colombia, 2015

**Factors**	**Factor 2**	**Factor 3**	**Factor 4**
**Factor 1**	**0.67**	**0.31**	**0.57**
**Factor 2**		**0.59**	**0.57**
**Factor 3**			**0.50**

When Cronbach’s alpha and Mc Donald’s Omega tests were applied, the coefficients indicated a reliability of 0.80 and 0.97, respectively, considering the complete 48-item scale and its division per factor, with a confidence interval for α ranging between 0.742 and 0.818. The individual coefficients of each item were superior to 0.75 in both tests. 

The cut-off point that revealed to be most consistent was 3.2, according to the ROC curve, indicating 66.7% sensitivity, 56.6% specificity and a positive prediction capacity of 72.6% to measure the attitude construct.

## Discussion

The objective in this study was to validate the Spanish version of the EAFAA when applied to Spanish speaking nurses in the Colombian context, demonstrating that 75% of the four component factors and the factor loading of the 49 items resulting from the content validation process^16^ presented adequate correlation coefficients (98% of the correlations between the factors ranged between moderate and strong), indicating a satisfactory representation of the construct measured, in line with the studies developed in Brazil, whose results indicated significant correlations between 75% and 80% of the factors, suggesting that this distribution indicates that evidence exists of a consistent factorial structure. 

After concluding the analysis of the factor loadings resulting from the confirmatory factor analysis, one item was excluded: 45: “Individuals with alcohol use disorders cooperate with their treatment”, because it did not reach the cut-off point permitted (0.30)^17^. This fact can be associated with the cultural differences and the approach used in care for this population in Colombia, being centered on internment, which presupposes the user’s unconditional cooperation in the treatment. The exclusion of this item was analyzed in detail to guarantee that it would not affect the reliability of the scale, as verified by means of Cronbach’s alpha and Mc Donald’s Omega, which indicated the existence of a strong correlation among the 48 remaining items.

Although Cronbach’s alpha is the most used technique in this kind of studies, some authors^18^ signal that this reliability ratio is not immune to limitations and questioning and, therefore, another technique was used with the same objective, which is to verify the reliability or internal consistency of the EAFAA. Mc Donald’s Omega test showed a reliability ratio of 0.97 for the complete version of the EAFAA - Spanish version, and coefficients superior to 0.8 when calculated for the individual items.

The results of the reliability coefficients of the EAFAA - Spanish version are consistent with the results presented in the primary original 96-item version (α=0.90)^12^, and in later studies intended to improve the scale in Brazil, with coefficients of α = 0.90 for an 83-item version^14^ and α = 0.89 for the current 50-item version^13^. These data indicate evidence to sustain the reliability of the EAFAA and its stability when reproduced in other languages and contexts, turning the scale increasingly robust to measure the attitudes towards alcohol, alcoholism and individuals with alcohol use disorders, suggesting the validation of its structural and metric equivalence^20^. 

The cut-off point that appointed the highest sensitivity and specificity of the EAFAA - Spanish version was 3.2, which indicates that higher coefficients can identify signs of positive attitudes, while lower coefficients can identify signs of negative attitudes of the population towards alcohol, alcoholism and individuals with alcohol use disorders. 

These results are similar to those reported in studies developed in Brazil^14^ ^-^ ^15^, which validated the scale among health professionals and nursing students, in which the cut-off points corresponded to 3.15 and 3.2, respectively, which refers to the capacity of the scale to precisely identify the positive and negative attitudes when applied in different contexts, including Spanish speaking nursing professionals. 

The validation of the EAFAA in Spanish, with a robust factorial structure and proven reliability, represents a knowledge advance, providing a reliable scale for use in that language. This study can support future research to assess the attitudes of health professionals in Spanish speaking populations, favoring the acknowledgement of differences and similarities of the attitudes among the Latin American cultures.

The use of a standardized tool can offer more reliable results, independently of the culture, supporting the construction of a body of knowledge about these professionals’ attitudes, which is still incipient in Latin America. That contributes to the elaboration of curricular structures that are more concerned with an issue that receives little value in the contemporary curricula, and which is fundamental to prepare health professionals in the area. 

In addition, the EAFAA demonstrated positive prediction capacities, which turns it into a useful tool for human resource management, through the identification of professional profiles based on their attitudes. Therefore, studies are needed to validate the use of the EAFAA in those scenarios.

### Study limitations

These study results indicate that the EAFAA is a valid and reliable scale for use in Spanish, although some limitations need to be taken into account: The study was developed in a specific region, limited to the specific characteristics of some Colombian cities, which can truly present cultural similarities with other regions of the country and the continent. Therefore, we suggest the repetition of this study in different populations and other Latin American regions, with a view to considering the use of the Spanish version of the scale, with greater security.

## Conclusions

The validation process of the EAFAA - Spanish version suggested that the scale is a valid and reliable instrument with robust psychometric properties, capable of measuring nurses’ attitudes in the Colombian culture towards alcohol, alcoholism and individuals with alcohol use disorders.
